# 2-[(Dimethyl­amino)­methyl­idene]propane­dinitrile

**DOI:** 10.1107/S1600536813004960

**Published:** 2013-02-23

**Authors:** Rajni Kant, Vivek K. Gupta, Kamini Kapoor, D. R. Patil, Madhukar B. Deshmukh

**Affiliations:** a X-ray Crystallography Laboratory, Post-Graduate Department of Physics & Electronics, University of Jammu, Jammu Tawi 180 006, India; b Department of Chemistry, Shivaji University, Kolhapur 416 004, India

## Abstract

In the title moleclue, C_6_H_7_N_3_, the mean plane of the dimethyl­amino group [maximum deviation = 0.006 (2) Å] forms a dihedral angle of 7.95 (18)° with the mean plane of the propane­dinitrile fragment [maximum deviation = 0.008 (2) Å]. In the crystal, weak C—H⋯N hydrogen bonds link the mol­ecules into a three-dimensional network.

## Related literature

For applications of enamines, see: Omran *et al.* (1997 ▶); Saleh *et al.* (1999 ▶). For related structures, see: Kant *et al.* (2012 ▶); Karlsen *et al.* (2002 ▶).
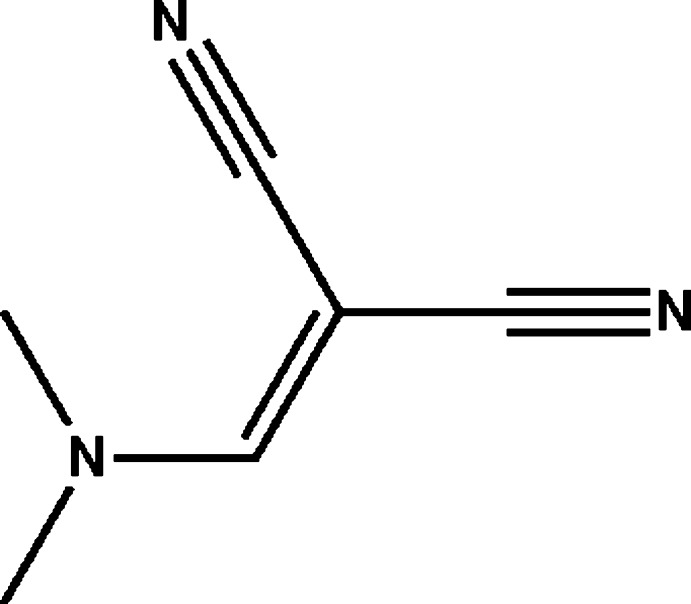


## Experimental

### 

#### Crystal data

C_6_H_7_N_3_
*M* *_r_* = 121.15
Monoclinic, 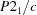

*a* = 4.0368 (3) Å
*b* = 15.5642 (10) Å
*c* = 10.8500 (7) Å
β = 97.488 (6)°
*V* = 675.89 (8) Å^3^
*Z* = 4
Mo *K*α radiation
μ = 0.08 mm^−1^
*T* = 293 K
0.3 × 0.2 × 0.2 mm

#### Data collection

Oxford Diffraction Xcalibur Sapphire3 diffractometer
Absorption correction: multi-scan (*CrysAlis PRO*; Oxford Diffraction, 2010 ▶) *T*_min_ = 0.637, *T*_max_ = 1.000
15029 measured reflections
1320 independent reflections
875 reflections with *I* > 2σ(*I*)
*R*_int_ = 0.067

#### Refinement

*R*[*F* ^2^ > 2σ(*F* ^2^)] = 0.066
*wR*(*F* ^2^) = 0.206
*S* = 1.05
1320 reflections
84 parameters
H-atom parameters constrained
Δρ_max_ = 0.23 e Å^−3^
Δρ_min_ = −0.16 e Å^−3^

### 

Data collection: *CrysAlis PRO* (Oxford Diffraction, 2010 ▶); cell refinement: *CrysAlis PRO*; data reduction: *CrysAlis PRO*; program(s) used to solve structure: *SHELXS97* (Sheldrick, 2008 ▶); program(s) used to refine structure: *SHELXL97* (Sheldrick, 2008 ▶); molecular graphics: *PLATON* (Spek, 2009 ▶); software used to prepare material for publication: *PLATON*.

## Supplementary Material

Crystal structure: contains datablock(s) I, global. DOI: 10.1107/S1600536813004960/lh5587sup1.cif

Structure factors: contains datablock(s) I. DOI: 10.1107/S1600536813004960/lh5587Isup2.hkl

Supplementary material file. DOI: 10.1107/S1600536813004960/lh5587Isup3.cml

Additional supplementary materials:  crystallographic information; 3D view; checkCIF report

## Figures and Tables

**Table 1:** Hydrogen-bond geometry (Å, °)

*D*—H⋯*A*	*D*—H	H⋯*A*	*D*⋯*A*	*D*—H⋯*A*
C2—H2⋯N8^i^	0.93	2.51	3.399 (4)	161
C4—H4*B*⋯N9^ii^	0.96	2.62	3.569 (4)	170

Symmetry codes: (i) 
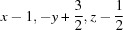
; (ii) 
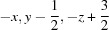
.

**Fig. 1.**
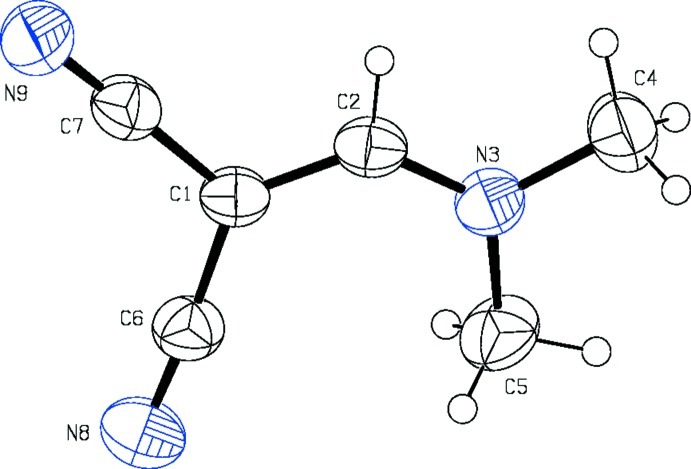
The molecular structure of (I) with displacement ellipsoids drawn at the 40% probability level. H atoms are shown as small spheres of arbitrary radii.

**Fig. 2.**
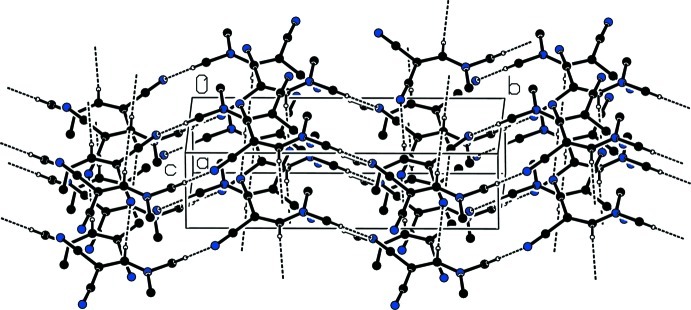
Part of the crystal structure with dashed lines showing weak intermolecular C—H···N hydrogen bonds.

## supplementary crystallographic information

### Comment

[(Dimethylamino)methylidene]propanedinitrile (I) is a potentially versatile substance which can be used for the synthesis of number of heterocyclic compounds and drug intermediates (Omran *et al.*, 1997; Saleh *et al.*, 1999).

In (I)(Fig.1), all bond lengths and angles are normal and correspond to those observed in related structures (Kant *et al.*, 2012; Karlsen *et al.*, 2002). The dihedral angle between dimethylamino group (N3/C2/C4/C5 with a maximum deviation of 0.006 (2)Å for N3) and propanedinitrile fragment (C1/C6/C7/N8/N9 with a maximum deviation of 0.008 (2)Å for C6) is 7.95 (18)°. In the crystal, weak C2—H2···N8^i^ and C4—H4B···N9^ii^ hydrogen bonds link molecules to form a three-dimensional supramolecular structure (Fig. 2, Table 1.).

### Experimental

In a 50 ml round bottomed flask charged with 3 mmol of malononitrile and 3 mmol of dimethyl formamide dimethylacetal was stirred for 2 - 3hrs at room temp. The reaction was monitored by TLC. After completion of the reaction, a precipitate was formed. Finally, the product was filtered and washed with pet ether. Yield: 75%, m.p. 361–363 K. Diffraction quality single crystals were grown by slow evaporation of an ethanol solution of the title compound at room temperature

### Refinement

All H atoms were positioned geometrically and were treated as riding on their parent C atoms, with C—H distances of 0.93–0.96 Å and with *U*_iso_(H) = 1.2*U*_eq_(C) or 1.5*U*_eq_(methyl C).

### Crystal data

**Table:**

C_6_H_7_N_3_	*F*(000) = 256
*M**_r_* = 121.15	*D*_x_ = 1.191 Mg m^−^^3^
Monoclinic, *P*2_1_/*c*	Mo *K*α radiation, λ = 0.71073 Å
Hall symbol: -P 2ybc	Cell parameters from 3950 reflections
*a* = 4.0368 (3) Å	θ = 3.8–29.2°
*b* = 15.5642 (10) Å	µ = 0.08 mm^−^^1^
*c* = 10.8500 (7) Å	*T* = 293 K
β = 97.488 (6)°	Block, colourless
*V* = 675.89 (8) Å^3^	0.3 × 0.2 × 0.2 mm
*Z* = 4	

### Data collection

**Table:**

Oxford Diffraction Xcalibur Sapphire3 diffractometer	1320 independent reflections
Radiation source: fine-focus sealed tube	875 reflections with *I* > 2σ(*I*)
Graphite monochromator	*R*_int_ = 0.067
Detector resolution: 16.1049 pixels mm^-1^	θ_max_ = 26.0°, θ_min_ = 3.8°
ω scans	*h* = −4→4
Absorption correction: multi-scan (*CrysAlis PRO*; Oxford Diffraction, 2010)	*k* = −19→19
*T*_min_ = 0.637, *T*_max_ = 1.000	*l* = −13→13
15029 measured reflections	

### Refinement

**Table:**

Refinement on *F*^2^	Primary atom site location: structure-invariant direct methods
Least-squares matrix: full	Secondary atom site location: difference Fourier map
*R*[*F*^2^ > 2σ(*F*^2^)] = 0.066	Hydrogen site location: inferred from neighbouring sites
*wR*(*F*^2^) = 0.206	H-atom parameters constrained
*S* = 1.05	*w* = 1/[σ^2^(*F*_o_^2^) + (0.1075*P*)^2^ + 0.0919*P*] where *P* = (*F*_o_^2^ + 2*F*_c_^2^)/3
1320 reflections	(Δ/σ)_max_ = 0.001
84 parameters	Δρ_max_ = 0.23 e Å^−^^3^
0 restraints	Δρ_min_ = −0.16 e Å^−^^3^

### Special details

**Table:**

Experimental. CrysAlisPro, Oxford Diffraction Ltd., Version 1.171.34.40 (release 27-08-2010 CrysAlis171 .NET) (compiled Aug 27 2010,11:50:40) Empirical absorption correction using spherical harmonics, implemented in SCALE3 ABSPACK scaling algorithm.
Geometry. All esds (except the esd in the dihedral angle between two l.s. planes) are estimated using the full covariance matrix. The cell esds are taken into account individually in the estimation of esds in distances, angles and torsion angles; correlations between esds in cell parameters are only used when they are defined by crystal symmetry. An approximate (isotropic) treatment of cell esds is used for estimating esds involving l.s. planes.
Refinement. Refinement of F^2^ against ALL reflections. The weighted R-factor wR and goodness of fit S are based on F^2^, conventional R-factors R are based on F, with F set to zero for negative F^2^. The threshold expression of F^2^ > 2sigma(F^2^) is used only for calculating R-factors(gt) etc. and is not relevant to the choice of reflections for refinement. R-factors based on F^2^ are statistically about twice as large as those based on F, and R- factors based on ALL data will be even larger.

### Fractional atomic coordinates and isotropic or equivalent isotropic displacement parameters (Å^2^)

**Table:**

	*x*	*y*	*z*	*U*_iso_*/*U*_eq_	
C1	0.3330 (6)	0.77364 (16)	0.7562 (2)	0.0501 (7)	
C2	0.2937 (6)	0.69295 (15)	0.7036 (2)	0.0510 (7)	
H2	0.1698	0.6914	0.6251	0.061*	
N3	0.4033 (5)	0.61815 (13)	0.74689 (19)	0.0565 (6)	
C4	0.3449 (7)	0.54179 (18)	0.6690 (3)	0.0752 (9)	
H4A	0.2019	0.5562	0.5941	0.113*	
H4B	0.2401	0.4983	0.7132	0.113*	
H4C	0.5542	0.5206	0.6483	0.113*	
C5	0.5891 (7)	0.6056 (2)	0.8695 (3)	0.0726 (9)	
H5A	0.7939	0.6377	0.8759	0.109*	
H5B	0.6386	0.5457	0.8821	0.109*	
H5C	0.4577	0.6253	0.9316	0.109*	
C6	0.5281 (6)	0.79802 (16)	0.8689 (2)	0.0568 (7)	
C7	0.1665 (6)	0.84244 (17)	0.6873 (2)	0.0578 (7)	
N8	0.6838 (6)	0.82284 (18)	0.9572 (2)	0.0805 (8)	
N9	0.0355 (6)	0.89864 (16)	0.6341 (2)	0.0789 (8)	

### Atomic displacement parameters (Å^2^)

**Table:**

	*U*^11^	*U*^22^	*U*^33^	*U*^12^	*U*^13^	*U*^23^
C1	0.0514 (13)	0.0581 (15)	0.0392 (13)	−0.0003 (11)	0.0003 (10)	0.0009 (11)
C2	0.0508 (13)	0.0622 (17)	0.0391 (13)	−0.0035 (11)	0.0017 (10)	0.0040 (11)
N3	0.0647 (13)	0.0565 (13)	0.0466 (13)	0.0020 (10)	0.0009 (10)	0.0064 (10)
C4	0.090 (2)	0.0557 (17)	0.077 (2)	−0.0048 (14)	0.0003 (16)	−0.0040 (15)
C5	0.0853 (19)	0.0757 (19)	0.0537 (18)	0.0151 (15)	−0.0032 (14)	0.0126 (15)
C6	0.0573 (15)	0.0640 (17)	0.0477 (15)	0.0020 (12)	0.0016 (12)	−0.0023 (13)
C7	0.0643 (16)	0.0599 (16)	0.0469 (16)	−0.0022 (13)	−0.0018 (12)	−0.0053 (13)
N8	0.0883 (17)	0.0896 (19)	0.0576 (16)	−0.0012 (14)	−0.0125 (13)	−0.0126 (14)
N9	0.0944 (18)	0.0635 (15)	0.0717 (18)	0.0074 (13)	−0.0155 (14)	0.0030 (14)

### Geometric parameters (Å, º)

**Table:**

C1—C2	1.380 (3)	C4—H4B	0.9600
C1—C6	1.417 (3)	C4—H4C	0.9600
C1—C7	1.424 (3)	C5—H5A	0.9600
C2—N3	1.311 (3)	C5—H5B	0.9600
C2—H2	0.9300	C5—H5C	0.9600
N3—C5	1.453 (3)	C6—N8	1.143 (3)
N3—C4	1.460 (3)	C7—N9	1.139 (3)
C4—H4A	0.9600		
			
C2—C1—C6	128.4 (2)	N3—C4—H4C	109.5
C2—C1—C7	116.5 (2)	H4A—C4—H4C	109.5
C6—C1—C7	115.0 (2)	H4B—C4—H4C	109.5
N3—C2—C1	130.2 (2)	N3—C5—H5A	109.5
N3—C2—H2	114.9	N3—C5—H5B	109.5
C1—C2—H2	114.9	H5A—C5—H5B	109.5
C2—N3—C5	123.9 (2)	N3—C5—H5C	109.5
C2—N3—C4	119.6 (2)	H5A—C5—H5C	109.5
C5—N3—C4	116.5 (2)	H5B—C5—H5C	109.5
N3—C4—H4A	109.5	N8—C6—C1	175.8 (3)
N3—C4—H4B	109.5	N9—C7—C1	178.6 (3)
H4A—C4—H4B	109.5		
			
C6—C1—C2—N3	5.6 (4)	C1—C2—N3—C5	2.7 (4)
C7—C1—C2—N3	−176.8 (2)	C1—C2—N3—C4	−176.2 (3)

### Hydrogen-bond geometry (Å, º)

**Table:**

*D*—H···*A*	*D*—H	H···*A*	*D*···*A*	*D*—H···*A*
C2—H2···N8^i^	0.93	2.51	3.399 (4)	161
C4—H4*B*···N9^ii^	0.96	2.62	3.569 (4)	170

Symmetry codes: (i) *x*−1, −*y*+3/2, *z*−1/2; (ii) −*x*, *y*−1/2, −*z*+3/2.
